# Unilateral Anterior Ischemic Optic Neuropathy: Chromatic Pupillometry in Affected, Fellow Non-Affected and Healthy Control Eyes

**DOI:** 10.3389/fneur.2013.00052

**Published:** 2013-05-10

**Authors:** Kristina Herbst, Birgit Sander, Henrik Lund-Andersen, Marianne Wegener, Jens Hannibal, Dan Milea

**Affiliations:** ^1^Department of Ophthalmology, Glostrup HospitalCopenhagen, Denmark; ^2^Department of Clinical Biochemistry, Bispebjerg HospitalCopenhagen, Denmark; ^3^Department of Ophthalmology, University Hospital AngersAngers, France; ^4^Singapore National Eye Centre, Singapore Eye Research InstituteSingapore; ^5^Neuroscience and Behavioral Disorders, Duke-NUSSingapore

**Keywords:** melanopsin, intrinsically photosensitive ganglion cells, ischemic optic neuropathy, pupillometry, pupil light response

## Abstract

The intrinsically photosensitive retinal ganglion cells (ipRGCs) express the photopigment melanopsin, which is sensitive to blue light. Previous chromatic pupillometry studies have shown that the post-illumination response is considered an indicator of the melanopsin activation. The aim of this study was to investigate the ipRGC mediated pupil response in patients with a unilateral non-arteritic anterior ischemic optic neuropathy (NAION). Consensual pupil responses during and after exposure to continuous 20 s blue (470 nm) or red (660 nm) light of high intensity (300 cd/m^2^) were recorded in each eye for 10 patients. Comparisons were performed both intra-individually (affected versus non-affected eyes) and inter-individually (compared with healthy controls). The pupil response was calculated both during the illumination and during the post-illumination phase. The pupil responses to blue and red colors were significantly reduced in the NAION-affected eyes, compared with the fellow non-affected eyes. When comparing the affected eyes with the healthy control eyes, the post-illumination responses were not significantly different. In addition, the post-illumination pupil responses after blue light exposure were increased in the fellow non-affected patients’ eyes, compared with the healthy controls. However, significance was only reached for the late post-illumination response. In conclusion, chromatic pupillometry disclosed reduced post-illumination pupil responses in the NAION-affected eyes, compared with the non-affected fellow eyes, suggesting dysfunction of the ipRGCs. Compared with the responses of the healthy controls, the blue light post-illumination pupil responses were similar in the affected eyes and increased in the fellow non-affected eyes. This suggests a possible adaptive phenomenon, involving the ipRGCs of both eyes after unilateral NAION.

## Introduction

Pupil light responses are mediated by the intrinsically photosensitive retinal ganglion cells (ipRGCs). The ipRGC contains the photopigment melanopsin and projects to the pupillomotor center *olivary pretectal nucleus* in the midbrain. Pupillometric measurements have been shown to be a potential tool to assess ipRGC function *in vivo* in humans (Gamlin et al., 2007; Kardon et al., 2009; Feigl et al., 2011; Kankipati et al., 2011; Park et al., 2011; Herbst et al., 2012; Léon et al., 2012).

*In vitro* primate cell recordings have shown that ipRGCs exhibit sensitivity to short wavelength light (480 nm, blue) of high photopic intensity and display a unique sustained cell firing even after light offset (Dacey et al., 2005). The sustained post-illumination pupil response after stimulation with blue light is considered an indicator of melanopsin activity (Gamlin et al., 2007). As the ipRGCs also integrate information from rods (stimulated by dim blue light) and cones (stimulated by longer wavelengths) (Dacey et al., 2005; Güler et al., 2008), the pupil response reflects both melanopsin and classical photoreceptor activation, based on the stimulus conditions (Kardon et al., 2009, 2011; McDougal and Gamlin, 2010; Park et al., 2011; Gooley et al., 2012; Léon et al., 2012).

Recent studies have suggested that outer photoreceptor diseases impair pupil re-dilation after exposure to bright blue light, delaying the time of recovery to the baseline size, while in patients with ischemic optic neuropathies, pupil re-dilation features are similar to those in healthy controls (Léon et al., 2012). In other bilateral optic neuropathies (advanced glaucoma), the blue light post-illumination pupil responses are reduced, as a result of the ipRGC lost (Feigl et al., 2011; Kankipati et al., 2011).

In order to further investigate the pupil responses in optic neuropathies using chromatic pupillometry, we compared pupil responses in patients affected by a strictly unilateral non-arteritic anterior ischemic optic neuropathy (NAION) intra-individually (affected eye versus fellow non-affected eye) and inter-individually (versus an age-matched healthy group).

## Materials and Methods

### Patients

We included patients with a typical initial clinical presentation of unilateral NAION (i.e., acute onset of painless, isolated visual impairment and visual field defect, associated with optic disk edema), where there was no evidence for another cause of optic neuropathy and no clinical signs of any associated retinopathy. In all the patients, an extensive work-up, including appropriate neuroimaging, allowed us to rule out other compressive, inflammatory, infectious, hereditary or toxic causes of optic neuropathy. All the patients were diagnosed and referred by experienced neuro-ophthalmologists at the Department of Ophthalmology, Glostrup University Hospital, Copenhagen, Denmark. Besides a complete neuro-ophthalmologic examination, all the patients underwent an evaluation of predisposing systemic vascular and local anatomical risk factors (disk at risk in the non-affected fellow eye) for NAION. The exclusion criteria were: a history of glaucoma, isolated raised intraocular pressure (IOP), cataracts, retinopathies, associated ophthalmologic conditions interfering with the pupil light responses or their recordings such as congenital or acquired iris defects, anterior segment abnormalities, and ptosis. Patients with diabetes or psychiatric disorders were also excluded, as were patients taking medication known to affect the pupil light response. An age-matched group of healthy individuals with normal vision was also included in the study. The healthy controls were explored between April and August, while the NAION patients were explored between August and September.

All included individuals (control healthy group and NAION patients) had the following evaluation: visual acuity measured on the ETDRS scale (the results being reported as decimal equivalent), color vision (Farnsworth D-15 Hue), visual fields (Humphrey, SITA standard 30-2 program, Zeiss, San Leandro, CA, USA), relative afferent pupil defect (RAPD) search using swinging flashlight test, IOP measurement by Goldmann tonometer, fundoscopic evaluation and fundus photography. The presence of RAPD was graded using neutral density filters. After the fundoscopic evaluation of the optic disk atrophy, the thickness of the retinal nerve fiber layer (RNFL) was estimated by OCT (Cirrus HD-OCT, Carl Zeiss Meditec, Inc., Dublin, CA, USA) in each eye. Full field ERG (ffERG) was proposed to every patient in order to rule out an associated occult photoreceptor dysfunction.

Additionally, in all subjects, blue light transmission through the lens was measured with an ocular fluorometer (Fluotron, Mountain View, CA, USA), and transmission value of 1.0 corresponded to totally clear lens and full transmission, as described earlier (Broendsted et al., 2011; Herbst et al., 2012).

Written and verbal informed consent was obtained from all participants. This research was carried out in compliance with the Declaration of Helsinki (2008), developed by the World Medical Association (WMA). The protocol of this study was approved by the appropriate local ethics committee of the Copenhagen Region (De Videnskabsetiske Komiteer for Region Hovedstaden, H-A-2009-033).

### Chromatic pupillometry

The consensual pupil light reflex (PLR) to a standardized light stimulation was recorded using a prototype monocular chromatic pupillometer (IdeaMedical, Copenhagen, Denmark) (Herbst et al., 2011, 2012) with an inbuilt light source consisting of monochromatic blue (narrow bandwidth LED, 470 nm, with 20–22 nm full width at half maximum) or red light (narrow bandwidth LED, 660 nm wavelength, with 20–22 nm full width at half maximum). The custom made light source consisted of a metal tube (diameter 4.4 cm), which projected the light onto a plastic hemisphere diffuser. The tube with diffuser was placed very close to the subject’s eye in contact with the inferior orbital rim: the viewing distance was approximately 5 cm (for details, see Herbst et al., 2011).

Light color and timing were controlled by a computer. The luminance output of the light stimulus was calibrated initially by a spectrophotometer (PR-655, Photo Research, Chatsworth, CA, USA) and re-checked before each session at a fixed distance (corresponding to the patient’s cornea) and angle. The light calibration was performed before each examination throughout the study to ensure equal conditions for the healthy subjects and the patient group, despite a seasonal difference at inclusion. The pupil size was continuously monitored and recorded using an infrared camera.

In the group of NAION patients, the pupil response of each eye was assessed using two successive recordings. Illumination with either red or blue equiluminant light at an intensity of 300 cd/m^2^ lasted for 20 s. The red (660 nm) light stimulus at this spectral range and photopic level is assumed to reflect mainly L/M cone activation (Dacey et al., 2005). Stimulation with blue light (470 nm) at high photopic level (300 cd/m^2^ light intensity) was used in order to preferentially activate the melanopsin containing ipRGCs and to detect the post-illumination response at this light level (Dacey et al., 2005; Gamlin et al., 2007). Prior to the recordings, each subject was instructed to relax during the pupil recording, not to move head or eyes, to avoid blinking, and to look straight forward, viewing an imaginary distant target in order to reduce accommodation. As described above, the recorded eye movements and position were monitored in real time by a trained observer via the inbuilt pupillometer window.

Each subject was placed in a quiet, dark room (under mesopic illumination, i.e., approximately 0.74 cd/m^2^) for approximately 5 min before the pupil testing. The ambient light was then turned off (0 cd/m^2^), and the patient was dark-adapted for 1 min prior to the pupil recording session.

Thereafter the continuous recording was initiated: during the first 10 s in darkness, *a mean baseline pupil diameter* was calculated, in order to normalize pupil size readings (See Data normalization and processing). Thereafter a continuous 20 s light stimulus (either blue or red light) was presented, and the pupil dynamics were recorded during the illumination and 1 min after light termination. The light stimulation protocol was standardized, stimulation with red light being followed by stimulation with blue light. A 3-min pause in mesopic light separated two successive recordings (i.e., red light test from blue light test), followed by a 1-min adaptation in prestimulus darkness before the next recording. By convention, chromatic pupillometry was performed only in one eye in each healthy control subject.

The dark-adapted baseline pupil size (BS) was measured after the initial 1 min dark adaptation (just before the recording procedure, described above). The arbitrary pupil size measurement, provided by the pupillometer, was converted to the diameter in mm, based on an artificial pupil size nomogram (Herbst et al., 2012). Due to the consensual approach, the BS was read from the non-affected eye and was used for normalization of the affected eye’s pupil responses, and vice versa.

### Data normalization and processing

Pupil data were collected, stored, and analyzed, as previously described in detail (Herbst et al., 2011, 2012). In brief, data were sampled at 20 Hz and smoothed using a nearest neighbor approach, i.e., each data point was compared with the next point and recalculated as the mean of the start point and the three adjacent points to each side. The inbuilt algorithm was used for evaluation of blink artifacts: in the case of artifact, all data points within 1 s were substituted by the values generated by a linear regression line, which was calculated from the point just before the blink to the first point of the next second.

**Figure 1:**
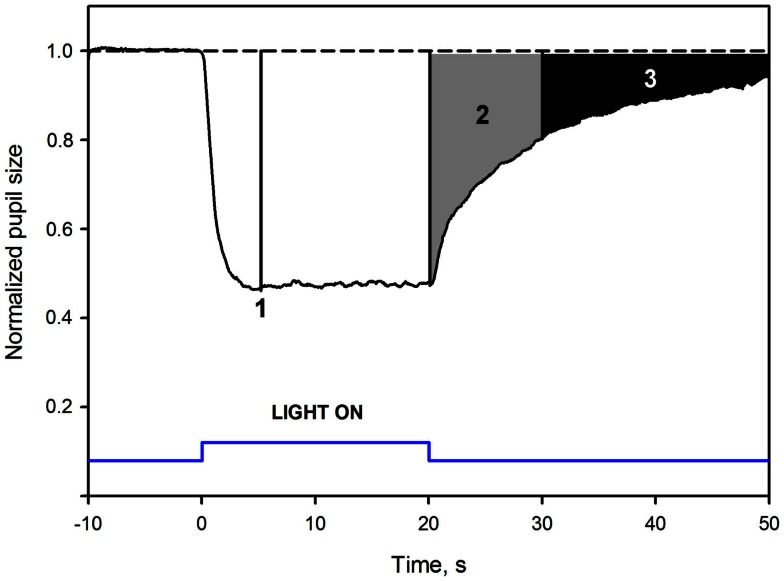
**Mean pupil response to blue 300 cd/m^2^ light in healthy subjects: pupil response parameters**. The dotted line at the top is an extrapolation from baseline pupil size. The *Y*-axis represents values of the normalized pupil size (NPS). On the *X*-axis, time −10 is the start of pupil video recording while in darkness. Time 0 is the onset of a 20 s, continuous, blue light stimulus (vertical bars at the bottom). Point 1 shows where the maximal contraction occurs (the maximal CA). The second parameter, 2, is the early post-illumination AUC (gray shaded area in the pupillogram), which is the summed pupil response amplitudes over 10 s after light termination. The late post-illumination AUC, 3 (black shaded area), another summed response parameter from 10th to 30th second after light termination. For parameter definitions, Section “Materials and Methods.”

To adjust for differences in *baseline pupil* between subjects, due to inter-individual variability, pupil response was expressed relatively to baseline pupil (normalized), similarly to the procedure used in other studies (Gamlin et al., 2007; Kardon et al., 2009; Kankipati et al., 2010; Park et al., 2011). Baseline pupil diameter was the average pupil diameter over 10 s in darkness before illumination (described above). Normalized pupil size (NPS) was defined as a ratio between the absolute pupil size and the average BS. After the normalization procedure, the BS was defined as 1.0 (Figure 1).

### Main outcome parameters

The maximal pupil contraction amplitude (maximal CA) was determined at the maximal contraction point within the first 6 s during illumination. This parameter reflected outer photoreceptor function (McDougal and Gamlin, 2010; Gooley et al., 2012). The maximal CA was calculated as the mean of three data points and reported as the maximal difference (expressed as %) from the baseline.

#### Post-illumination area under the curve

An area under the curve (AUC) was defined as the summed pupil amplitudes over time, obtained from the pupil response curve (Herbst et al., 2011, 2012, Figure 1). The area was calculated as: $\text{AUC}=\left(\Sigma_{t0}^{t1}1.0-\text{NPS}\right)$, where *t*_0_ was the starting time point of pupil response summation, *t*_1_ the stopping time point of summation, 1.0 was the baseline pupil, and NPS was the normalized pupil size. The unit of the AUC is NPS*time.

Two post-illumination AUCs were calculated: *early AUC* during an early post-illumination phase (0–10 s after the light offset) and *late AUC* during a late post-illumination phase (10–30 s after the light offset, Figure 1). The rapid pupil re-dilation (first seconds after light offset) of the early AUC is considered to include contributions from both rods and S-cones, modulating the activity of the ipRGCs (Lall et al., 2010; McDougal and Gamlin, 2010; Allen et al., 2011), while the late AUC probably only reflects the melanopsin activity.

An example of a waveform pupillogram and the measured pupil response parameters is illustrated in Figure 1.

### Statistical analysis

Statistical Analysis System (SAS) software (SAS version 9.2, SAS Institute Inc., Cary, NC, USA) was used for statistical analysis. Data was analyzed using a linear model, including the dark-adapted pupil diameter in absolute values (mm), gender and age as covariates, and outcome parameters were adjusted by the Tukey-Crammer method for multiple comparisons. Due to a small sample size, the main outcome results were also confirmed with non-parametric tests. A *p*-value < 0.05 was considered significant for all tests. For calculations of visual acuity, ETDRS units were used and converted to decimal equivalents in tables for easy overview.

## Results

Ten patients (median age 60 years, range 48–71 years) with typical, strictly unilateral NAION were included in the study. Unilateral visual impairment was found in all the patients: in four patients, visual acuity was equal or worse than counting fingers. The median visual acuity of the remaining six patients was 0.5 (range: 0.1–1.4). The median visual acuity of the non-affected fellow eyes of NAION patients was 1.10 (range: 1.0–1.7). The mean interval between the NAION occurrence and inclusion in the study was 38 months (95% CI: 20–56 months).

The healthy group included 11 age-matched individuals (median age = 61, ranged 47–66 years) with a mean visual acuity expressed as a decimal number of 1.40 (95% CI: 1.2–1.6). The visual acuity of the healthy controls and in the non-affected eyes of the NAION patients was comparable (*p* = 0.11). The demographic and the clinical findings of the patients at inclusion are summarized in Table 1.

**Table 1:** **Summary of the demographics and clinical findings of the NAION patients at inclusion**.

Pt. nr.	Sex	Age onset, years	NAION duration, years	VA aff. eye	VA non-aff. eye	C/D ratio	Risk factors	Disc edema at onset	Neuroimaging	VF defect
1	M	42	5	1.4	1.33	0.25	AH, HCh	Diffuse	+	Altitudinal
2	M	47	2	LP	0.96	0.1	AH, HCh	Diffuse	−	Central
3	M	53	1	0.2	1.46	0.2	AH, HCh	Diffuse	+	Altitudinal
4	M	57	2	0.1	1.92	<0.1		Diffuse	−	Altitudinal
5	M	53	8	0.7	1.01	0.1	AH	Diffuse	+	Arcuate
6	F	65	2	FC	1.10	0.1	AH, HCh	Diffuse	+	Cecocentral
7	M	67	1	0.9	1.46	0.1	AH	Segment	+	Nasal
8	F	65	4	NLP	1.10	<0.1	AH	NA	+	Central
9	F	67	4	HM	1.01	0.3	AH	Segment	+	Cecocentral
10	M	48	1	0.1	1.05	0.2	HCh	Diffuse	+	Altitudinal

*All ten NAION patients had a relative afferent pupillary defect (RAPD)*.

*VA, visual acuity (decimal equivalent converted from ETDRS); aff. eye, affected eye; non-aff. eye, non-affected eye; C/D ratio, cup/disk ratio in the non-affected eye; LP, light perception; CF, finger count; NLP, no light perception; HM, hand movements. Risk factors: AH, arterial hypertension; HCh, hypercholesterolemia. Neuroimaging defines CT and/or MRI*.

### Pupil responses in the affected eyes versus the non-affected fellow eyes in the NAION patients

**Figure 2:**
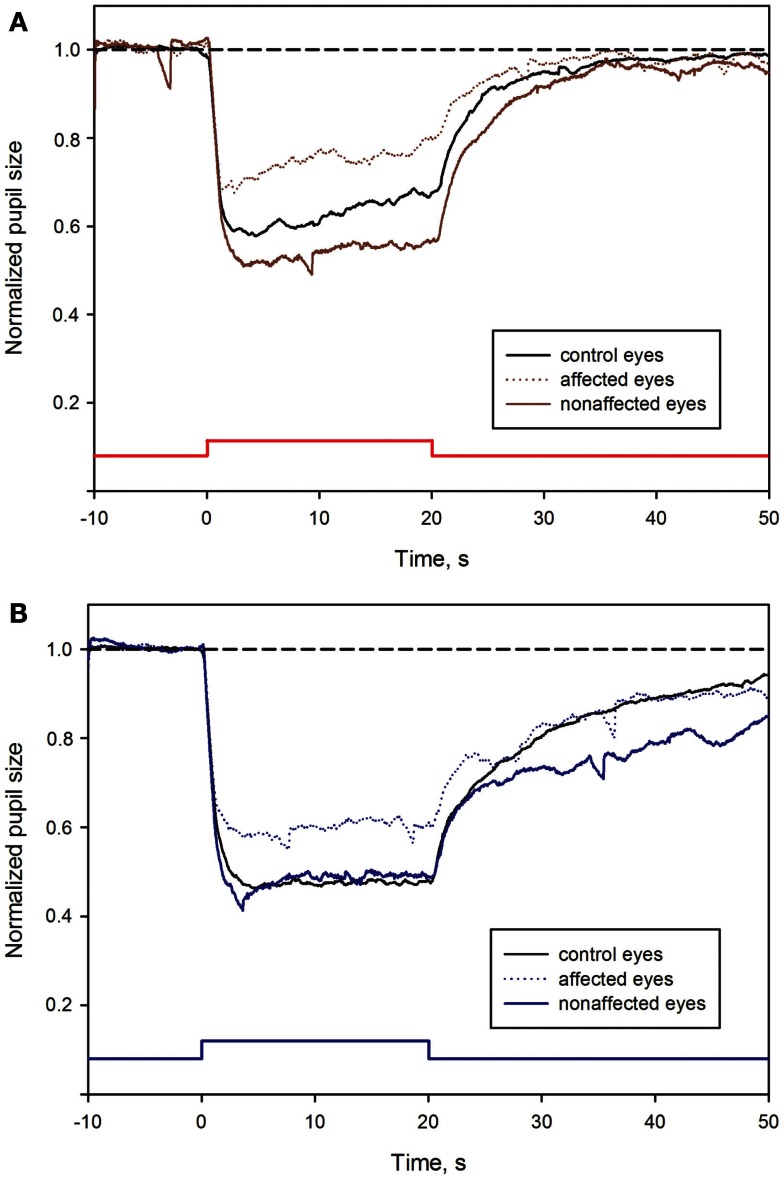
**Pupil responses in NAION-affected and fellow non-affected eyes, and in healthy control eyes**. **(A)**: pupil response curves for stimulation with red light. **(B)**: pupil response curves for stimulation with blue light. The broken line represents the reference line of baseline pupil size equal to 1.0. The vertical bar at the bottom indicates light stimulus onset: time 0 is light onset, time 20 is light offset. For parameter definitions, Section “Materials and Methods” and Figure 1. In all NAION patients, most of the pupil response parameters to 300 cd/m^2^ red **(A)** or blue light **(B)** were significantly reduced in the affected eyes (stippled curve), compared with the non-affected eyes (solid curve). When comparing the affected eyes with the healthy control eyes (black curve), the pupil responses during light exposure (maximal contraction amplitudes) were reduced in the affected eyes, reaching significance only at blue light conditions (*p* < 0.05, **B**). No significant difference was found *for the post-illumination response*. In contrast, *the blue light late post-illumination response* in the non-affected eyes was significantly increased (*p* < 0.05, **B**), compared with the healthy controls.

Most of the pupil response parameters, using blue or red light stimulation (maximal CA and early AUCs) were significantly reduced in the affected eyes (*p* < 0.05), compared with the fellow non-affected eyes. The only exceptions were the post-illumination late AUC after exposure to red light and the early AUC after exposure to blue light: they did not reach significance level (see Figure 2; Table 2).

**Table 2:** **The pupil responses to red and blue light for the affected eyes of patients with unilateral NAION, the non-affected eyes in the same patients and a group of age-matched, healthy controls**.

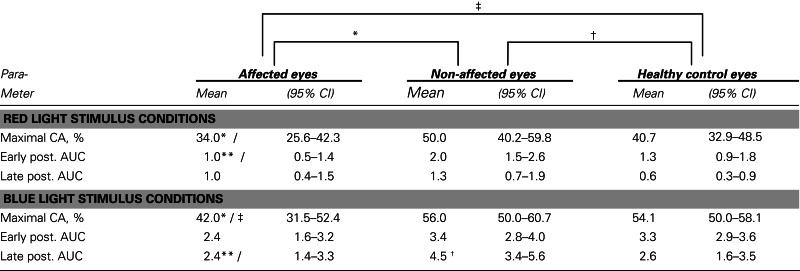

*Pupil response parameters are given as mean with a 95% confidence interval*.

*The pupil response was compared between the groups (NAION-affected eyes, fellow non-affected eyes in NAION patients, healthy control eyes), and *p-*values were adjusted for multiple comparisons by Tukey-Crammer method*.

Overall, the responses of *the affected eyes* were decreased compared with *the non-affected eyes* (significant changes indicated by *p < 0.05 and **p < 0.01). The responses of *the affected eyes* were also decreased compared with *the healthy controls*, though to a lesser degree and significant only for the maximal contraction amplitude (‡*p* < 0.05) to blue light. In contrast, the pupil responses were increased in the *non-affected eyes* versus *the healthy controls*, but significance was only reached for the late post-stimulus response for blue light (†*p* < 0.05).

**Maximal CA* (contraction amplitude) is the difference between the baseline pupil size (BS) and the normalized pupil size (NPS) at maximal contraction to light. *Areas under the curve (AUC): early*post-illumination AUC is the summed response amplitudes during the first 10 s after light termination. *Late* post-illumination AUC is the summed response within 10–30 s after light termination*.

Affected vs. non-affected eyes: *p < 0.05, **p < 0.01.

*Affected vs. control eyes: ‡*p* < 0.05*.

*Non-affected vs. control eyes: †*p* < 0.05*.

*CA, contraction amplitude; post. AUC, post-illumination area under the curve. For parameter definitions, see Materials and Methods and Figure 1*.

### Pupil responses in the affected eyes versus the healthy control eyes

During and after red light stimulation, no significant differences in pupil responses were found between the affected eyes in the NAION patients and the healthy control eyes (Figure 1; Table 2). The blue light pupil responses, obtained from the affected eyes, revealed a significant reduction of the maximal contraction amplitude, compared with the healthy controls (*p* < 0.05). However, the early and the late post-illumination AUCs in the affected eyes were not significantly different from those of the healthy control eyes (Figure 2; Table 2).

### Pupil responses in the non-affected fellow eyes of the NAION patients versus the healthy control eyes

A significant increase of the late post-illumination AUC after blue light stimulation (*p* < 0.05) was found in the non-affected patients’ eyes, when compared with the healthy controls. The red light pupil response parameters were numerically increased, but not significantly different between these two eye groups (Table 2).

### The dark-adapted (baseline) pupil diameter

The dark-adapted (baseline) pupil diameter (mm) was recorded prior to each test session. The mean dark-adapted (baseline) pupil size of the affected eyes was 6.37 mm (95% CI: 5.15–7.59 mm), and in the fellow non-affected eyes it was 6.43 mm (95% CI: 5.45–7.42 mm). The mean of the healthy control eyes was 7.03 mm (95% CI: 6.46–7.60 mm). There was no significant difference between the baseline pupil diameters in the three groups (*p* = 0.43). Moreover, in our study, the main outcome parameters were not influenced by the BS (data analyzed with linear models).

### The average RNFL thickness

The mean average RNFL thickness of the affected eyes was 56 μm (95% CI: 53–60 μm), which was significantly lower (*p* < 0.001) than the mean of the fellow non-affected eyes of 90 μm (95% CI: 82–97 μm) and the mean of the healthy control eyes of 86 μm (95% CI: 80–92 μm). The mean thickness of the healthy control eyes was not significantly different from that of the non-affected patients’ eyes (*p* = 0.09).

### Lens transmission for blue light

The results of the blue light transmission through the lens did not reveal any significant difference between the groups (*p* = 0.98): in the affected eyes, mean lens transmission was 0.66 (95% CI: 0.54–0.78), in the non-affected eyes it was 0.65 (95% CI: 0.51–0.79), and in the healthy control eyes it was 0.61 (95% CI: 0.55–0.68).

## Discussion

The first result of this study is that, in patients with a unilateral NAION, the pupil responses were significantly reduced in affected eyes, compared with the fellow non-affected eyes, both during the illumination and during the post-illumination phase. This is an expected result, consistent with the unilateral atrophy of the retinal ganglion cells, probably including the ipRGCs and their axons.

The second and unexpected result is that, despite the significant optic atrophy after unilateral NAION, there was no significant reduction of the post-illumination responses to blue light in the affected eyes, when compared with the healthy, age-matched population.

In addition, the late post-illumination pupil response after exposure to blue light was *increased* in the fellow non-affected eyes in NAION patients, when compared with healthy controls.

The finding that the post-illumination pupil responses in the NAION-affected eyes were not reduced, when compared with those recorded in the age-matched healthy controls, is in agreement with a previous report by Léon et al. (2012). In this study, the authors suggested that the intensity of the photic stimulus (100 cd/m^2^) was insufficient to stimulate the melanopsin photopigment contained by the ipRGCs. However, in our study, we assumed that the light intensity of 300 cd/m^2^ was high enough to evoke the ipRGC response, and a clear difference was seen *between* the eyes of the unilateral NAION patients. The second hypothesis is that the melanopsin-expressing ipRGCs could have been spared in the affected eyes, since they are thought to be very resistant to degeneration. However, intra-individual comparisons showed reduced pupil responses in the affected versus the non-affected eyes in patients, suggesting *a functional impairment* of the ipRGCs in the affected eyes. Third, we have to take into account the possibility of biased results by other factors, affecting the pupil response. Indeed, in our study, the mean size of the pupils at baseline was slightly larger in the healthy controls than in the patients, though not reaching statistical significance. Potentially, larger pupils could lead to an overestimation of the pupil response in the healthy controls, but if this is the case, the relative pupil response in the control eye would be larger than reported. Moreover, a previous report has suggested that a marked increase in pupil size would result in a modest increase in post-illumination response in blue light conditions (Nissen et al., 2011). Another possible bias is the fact that the patients and the healthy controls have been investigated during different seasons of the year, which could play a role (Thorne et al., 2009). Another bias could be the difference in pupil responses obtained via a direct versus a consensual approach. Previous reports using white pupillometry have shown a significantly larger direct pupil response (Carle et al., 2011), although consensual and direct pupil reflex are considered to be similar due to equal innervations via the Edinger-Westphal nucleus. Age-related lens changes could bias the pupil responses as well, although in our previous study, we found no relationship between lens changes related to age and the late post-illumination pupil response (Herbst et al., 2012). Moreover, the included patients and the age-matched healthy subjects did not have clinically significant cataracts, and this was also confirmed by lens transmission measurements. Other limitations of our study include its small sample size and the possible clinical variability between patients.

An unexpected finding of this study concerns the increased post-illumination pupil response in the fellow non-affected eyes, compared with the healthy controls. An adaptive upregulatory mechanism could explain such a process, affecting the blue light post-illumination pupil response in *both* eyes after unilateral NAION. Though speculative, such a phenomenon would explain the “normal” pupil response in the affected eye and the increased pupil response in the fellow eye, compared with the healthy, age-matched, control population. If such an adaptive process exists, it could occur at a central or/and peripheral (retinal) level. At the central level, a reduced unilateral ipRGC input could induce adaptive changes in the brainstem within the pupillomotor centers (the optic pretectal nuclei, OPN), which send *bilateral* fibers to the preganglionic neurons within the Edinger-Westphal nuclei. Similarly, it has been shown that removal of eyes *amplifies* a normally dampened endogenous circadian rhythm within the suprachiasmatic nucleus, SCN (Beaulé and Amir, 2003). At the retinal level, ipRGCs may undergo a local adaptive process via neurotransmitter modulating adaptation in the retina (Van Hook et al., 2012). In line with such a hypothesis, it has been shown that the expression of other opsins in the retina may be upregulated after unilateral optic nerve section in mice (Schremser and Williams, 1992).

The analysis of the pupil responses revealed a trend toward greater maximal contraction and toward greater partial post-illumination pupil contraction at red light conditions in the non-affected eyes than in the control eyes. Although the late post-illumination response to red light is less reliable than the response to blue light (Herbst et al., 2011), we cannot rule out an adaptive change at the central level, which may not be related to melanopsin mediated input.

Different causes of optic neuropathies may affect differently the functional integrity of the ipRGC system. Indeed, in humans, advanced glaucoma causes a significant decrease of the ipRGC mediated post-illumination pupil responses (Feigl et al., 2011; Kankipati et al., 2011). In contrast, a relatively preserved pupil response to blue light was reported in a patient with unilateral Leber’s hereditary optic neuropathy (Kawasaki et al., 2010), and partial preservation of fibers subserving pupil function was also described in a morphological study including patients with the same condition (Sadun et al., 2000). The very few, previous histo-pathological studies performed in human eyes with NAION (Tesser and Levin, 2003), did not look specifically at the number or the morphology of the remaining ipRGCs after the ischemic insult. Thus, further anatomo-clinical studies in NAION are needed, with the aim to correlate specific pupil responses with subsequent histological retinal findings in the same eyes, focusing in particular on the remaining ipRGCs.

In conclusion, the present chromatic pupillometry evaluation, performed in patients with unilateral NAION disclosed a significant decrease in the consensual pupil responses to blue and red light, when comparing the affected and the fellow non-affected eyes. When comparing responses of these patients with those of the healthy group, there was an unexpected comparable late post-illumination response in the affected eyes and an increased response in the non-affected eyes, suggesting an adaptive and possibly overall upregulated activity of the melanopsin system after unilateral NAION. In order to confirm these preliminary findings, further studies, using both open and closed loop pupillometry, are required.

## Conflict of Interest Statement

The authors declare that the research was conducted in the absence of any commercial or financial relationships that could be construed as a potential conflict of interest.
